# A Novel Vehicle Classification Using Embedded Strain Gauge Sensors

**DOI:** 10.3390/s8116952

**Published:** 2008-11-05

**Authors:** Wenbin Zhang, Qi Wang, Chunguang Suo

**Affiliations:** 1 Department of Automation of Testing and Control, Harbin Institute of Technology, Harbin, China, and Mailbox: 351; E-mail: wangqi@hit.edu.cn; 2 MEMS Center, Harbin Institute of Technology, Harbin, China; E-mail: suochunguang@126.com

**Keywords:** Vehicle classification, Embedded strain gauge sensor, Support vector machine, Multisensor data fusion

## Abstract

This paper presents a new vehicle classification and develops a traffic monitoring detector to provide reliable vehicle classification to aid traffic management systems. The basic principle of this approach is based on measuring the dynamic strain caused by vehicles across pavement to obtain the corresponding vehicle parameters – wheelbase and number of axles – to then accurately classify the vehicle. A system prototype with five embedded strain sensors was developed to validate the accuracy and effectiveness of the classification method. According to the special arrangement of the sensors and the different time a vehicle arrived at the sensors one can estimate the vehicle's speed accurately, corresponding to the estimated vehicle wheelbase and number of axles. Because of measurement errors and vehicle characteristics, there is a lot of overlap between vehicle wheelbase patterns. Therefore, directly setting up a fixed threshold for vehicle classification often leads to low-accuracy results. Using the machine learning pattern recognition method to deal with this problem is believed as one of the most effective tools. In this study, support vector machines (SVMs) were used to integrate the classification features extracted from the strain sensors to automatically classify vehicles into five types, ranging from small vehicles to combination trucks, along the lines of the Federal Highway Administration vehicle classification guide. Test bench and field experiments will be introduced in this paper. Two support vector machines classification algorithms (one-against-all, one-against-one) are used to classify single sensor data and multiple sensor combination data. Comparison of the two classification method results shows that the classification accuracy is very close using single data or multiple data. Our results indicate that using multiclass SVM-based fusion multiple sensor data significantly improves the results of a single sensor data, which is trained on the whole multisensor data set.

## 1. Introduction

Traffic data collected on highways have many applications, depending on the various agencies' needs. Traffic parameters which are the subject of direct measurement include the vehicle velocity, number of vehicles moving in the same direction, time distance between the vehicles, length of traffic jams, time of access to the traffic, etc. Very useful parameters are also the types (i.e. motor car, delivery van, lorry, trailer, etc.) and the number of moving vehicles belonging to the specified type. The type of vehicle is very important for the access control (areas closed to some vehicle types, limits of speed and weight for different vehicle types), statistical purposes and weighing of vehicles in motion.

Recently, a variety of methods have been proposed to identify vehicle class, weight and speed. For instance, vehicle parameters are derived from traffic-induced signals of Inductive Loop Detectors (ILDs) [1-4]. These detectors are imbedded in the roadway surface where heavy traffic and construction can cause damage to detectors. They have high failure rates due to poor maintenance [5]. High maintenance costs have caused agencies to seek replacement technologies. Based on recent advances in computer vision, image analysis techniques have also been applied to develop vehicle classification systems [6-10]. Under adverse real-world conditions, techniques based on image analysis are often affected by weather conditions such as rain and fog, low resolution/contrast and this often leads to low classification accuracy [11]. There are a number of other sensing technologies relevant to vehicle classification. These technologies include infrared, ultrasonic, radar, microwave, and video detectors. They each have their own strengths and weaknesses.

Pattern recognition is concerned with how to divide the classification of objects into categories. For the last 30 years, pattern recognition has been used with increasing success in a number of areas such as medicine, weather forecasting, automated industrial inspection and transportation. Vehicle classification is a type of pattern recognition, so many effective pattern recognition methods and combinations of multiple sensors have been used for classification and identification of vehicles in a particular vehicle classification system which have achieved satisfactory results, such as artificial neural networks [1, 4, 6, 11], Bayesian network data fusion [12], fuzzy data fusion [13], etc.

In this paper a novel pavement strain-based vehicle classification approach is developed, which may be used to provide pavement structural monitoring information. Using this approach, vehicles passing over a pavement deck can be classified purely from strain-response readings taken from the structure over which truck is traveling. A vehicle classification is usually determined according to particular countries' criteria with regards to a specific vehicle design feature: number of axles, distance between axles, etc. A vehicle's category parameters can be extracted from multiple strain response curves when a vehicle crosses the instrument-containing pavement. The accuracy of any classification scheme depends on the accuracy of the equipment used to capture the values of the discrimination variables and the accuracy of the corresponding classification algorithm(s). In order to be obtain vehicle classification parameters, a number of sensors will be used and the classification parameters of each are repeatedly measured by multiple sensors placed at different measurement locations.

In China, the types of vehicle vary from state to another, depending on the prevailing economic and social activities. At a state level, different activities also use of different vehicles in different areas, which leads to a lack of a uniform standard for the feature parameters of vehicles (axle number, axle distance), so establishing some fixed thresholds for classifying vehicles is very difficult, and at the same time the classification accuracy is not high. On the other hand, these patterns from different types of vehicles seemed to have a lot of overlap between them; this necessitates the use of pattern recognition and classification techniques to distinguish between vehicle groups. Good separation is that which results in minimum classification errors. In our research, a support vector machines (SVMs) machine learning method was employed to process feature vectors extracted from multiple strain time histories to obtain the vehicles' classification information. The method is still new and believed to be stronger in classification problems than neural networks, especially in their principles of problem generalization. The SVM uses structural risk minimization (SRM) that minimizes the upper bound on the expected risk and is said to be superior to neural network's empirical risk minimization (ERM) [14,15].

The main aim of this research was to investigate the feasibility of developing a novel sensor system based on multiple embedded strain gauges installed in the pavement to classify moving vehicles. In the following sections, the pavement test bench based on multiple strain gauges and data collection equipment is introduced, followed by results from a preliminary analysis. Thereafter, based on pavement dynamic strain response time histories subjected to the moving load vehicle classification approach are presented in detail, including information on the analysis framework, key feature extraction method, one-against-one multi-class SVMs development, analysis results and discussions.

## 2. Instrumental Pavement Test Bench and Strain-Vehicle Database

### 2.1 Description of the testing bench

An instrumental pavement panel (measuring 4.6 m in length and 4.5 m in width) was installed in 2004 along the roadway on k220+300 of Tong-Three state road, in Jiamusi of Heilongjiang province, Northeast China. This panel were built of concrete materials and instrumented to verify durability and potential for long-term usage. During 2005-2006, periodic measurements acquired pavement strain data as the basis for development of our strain-based vehicle classification.

**Figure 1.**
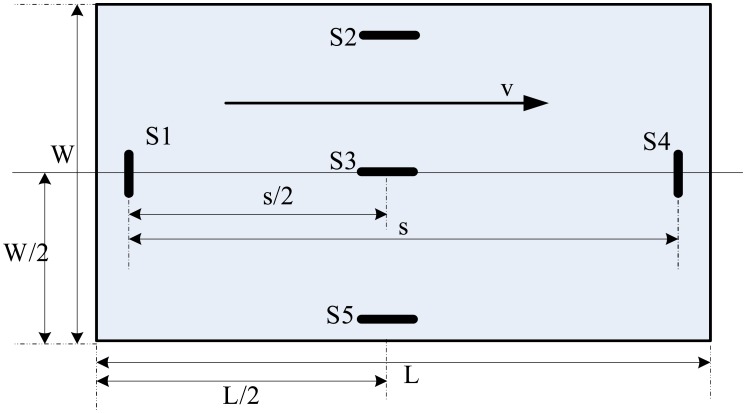
The layout plan of the strain gauge sensors.

**Figure 2.**
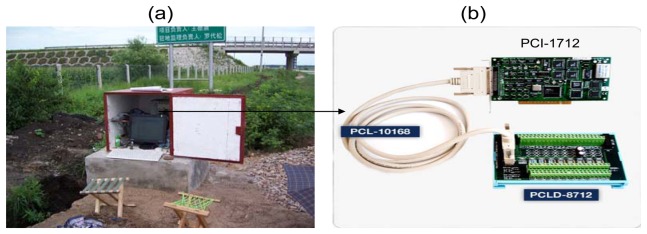
Photos of: (a) the data collection device (b) the data collection card and interface board.

The testing bench includes five embedded concrete strain gauge sensors located symmetrically below the surface of the pavement slab (Figure 1), used to measure the rigid pavement strain response. In this figure W =4.50 m is the pavement slab width; L =4.6 m is the pavement slab length; v is the direction of traffic; sensors S1, S2, S4, S5 are 25 cm under the edge of pavement; s=4.1 m is the distance between S1 and S4. This layout can be used to measure all the vehicles travelling on the right side of the road. Through the data acquisition system (this PCI-data acquisition board supporting 12 bit 16 single-ended or 8 differential analog inputs maximum up to 1 MHz sampling rate, made by ADVANTECH CO. LTD PCI-1712 Multifunction DAQ, refer to Figure 2), live data from the strain gauges and the artificial observations are synchronized and stored in a temporary buffer. The acquisition program, written in C++, provided for different sampling rates and times. A sampling rate of 2 kHz for 2.5 sec for each channel was found to be most appropriate for each vehicle.

As all the strain gauges are located on a westbound lane (Figure 1), this study only focuses on records of vehicles traveling in this direction. Furthermore, for this relative simple structural system, the strains recorded by the different gauges were found to be highly correlated. In view of this fact, only the strain data of sensors 1, 3, 4 were utilized in this study.

### 2.1 Pavement strain measurement

**Figure 3.**
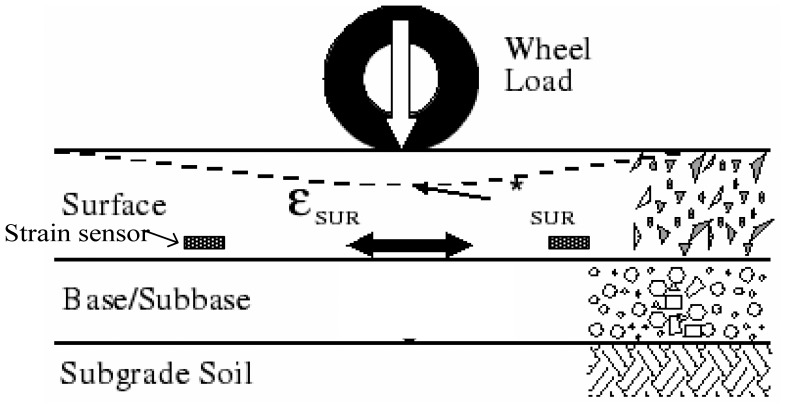
Illustration of the strain caused by moving wheel loads.

Strain sensors measure the expansion and contraction of pavement material due to mechanical stress. Like all transducers, these sensors rely on indirect measurement for determining strains. The strain gauge is embedded into the pavement surface (refer to Figure 3). Then, the strain *ε_SUR_* experienced by the pavement is transferred directly to the gauge, which responds with a linear change in electrical resistance.

### 2.2 Embedded Strain Gauge Sensor

**Figure 4.**
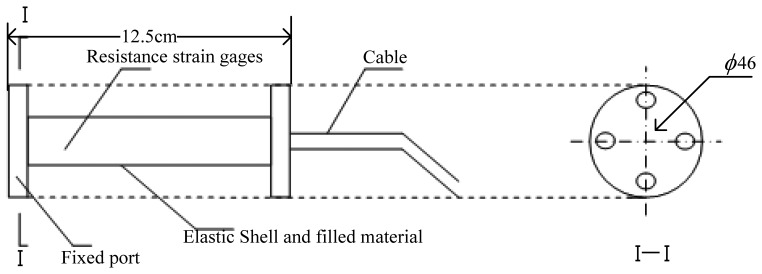
The structural sketch of the embedded strain gauge sensor.

**Figure 5.**
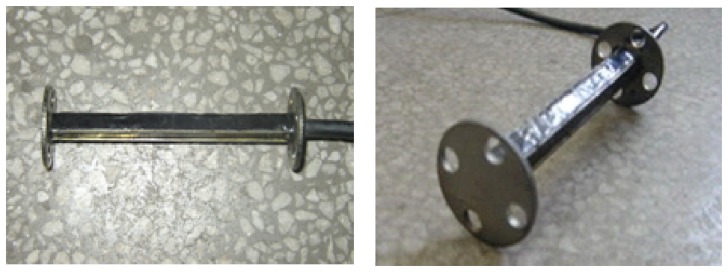
Photos of the embedded strain gauge sensor.

Two common gauge sensor types are the electrical resistance strain gauge and the fiber optic strain gauge. For selecting a electrical resistance strain gauge the main considerations are that they are relatively inexpensive, can achieve overall accuracy of better than ±0.1%, they are available in a short gauge length, are only moderately affected by temperature changes, have small physical size and low mass, and are highly sensitive. Resistance strain gauges can be used to measure both static and dynamic strains. Figures 4 and 5 show a structural sketch of the embedded concrete dynamic strain sensor and photos, respectively. The embedment strain gauges are designed for direct embedding in concrete. The dimensions of the strip strain sensors are 12.5 cm length, 1.2 cm width and 1 cm thickness. They are uniaxial embeddable strain gauges, with self-temperature-compensation, and a resistance of 350±0.5 Ω. The gauge factor is 2.0 and the modulus of elasticity is 30,000 Mpa. Its standard range is -1500 *με* (compressive strain) and +400 *με* (tensile strain). The frequency scope could be extending from 0 Hz to 3 kHz which is used for measuring dynamic strain response in the concrete pavement subjected to moving vehicular loads. It is extra rugged to resist bending, and with large flanges to provide a greater engagement area (Figure 5).

Since the embedded concrete strain gauge is tightly bonded with epoxy to the rigid pavement, the strain will be transferred to strain gauge. The piezoresistive material of the strain gauge will produce a relative resistance change. A Wheatstone bridge is usually employed to transfer the relative resistance change of piezoresistive gauges into voltage output. The dynamic strain meter will provide incentive power for Measurement Bridge and amplify the output voltage *V_out_*. When *V_out_* is measured, the quantity of strain may be calculated from Equation (1):

(1) $$\varepsilon_{\text{SUR}}=\frac{V_{\text{out}}K}{k_{0}}$$

where *ε_SUR_* is the longitudinal strain of the surface rigid pavement, *V_out_* is the output of the dynamic strain meter, *K* is the sensitivity of the strain meter (here *K*=200 *με*/1.2*V*), *k_0_* is the amplifier factor of the strain gauge (here *k_0_* =2).

### 2.3 Characteristics of Traffic-Induced Pavement Strain Response

**Figure 6.**
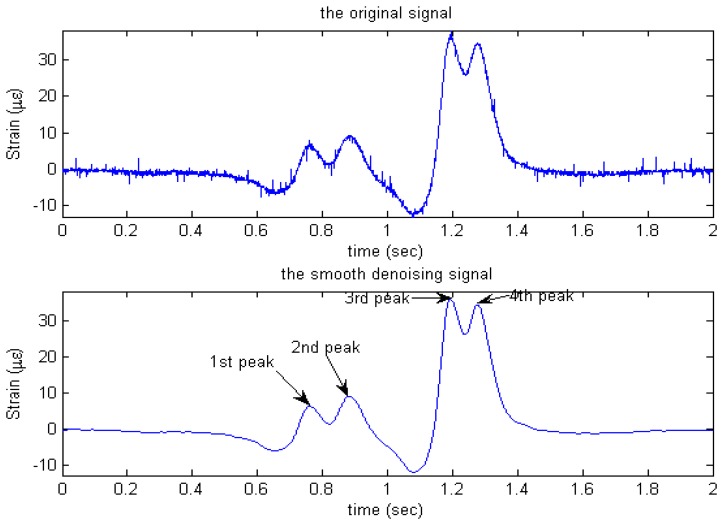
Characteristics of a four axle traffic-induced strain response time history.

Herein, the nature and characteristics of the recorded traffic-induced strain time histories are first investigated. Figure 6 shows a representative record corresponding to a 4-axle semi-trailer truck (1F+2M+1R, Table 1). When a vehicle axle crosses the pavement slab, the strain response increases significantly. In Figure 6, four major peaks can be seen in the time history, each corresponding to an axle. In addition, the space of these peaks is consistent with the distance between the axles. For instance, the 2^nd^ peak is far from the 3^rd^ peak as these axles are widely spaced, and the magnitude of each strain peak primarily depends on the axle weight. In the linear range of rigid pavement response, the stress vehicle-induced is proportional to the strain. i.e. *ε_SUR_* = *σ*/*E*, where *ε_SUR_* is the longitudinal strain induced by the vehicle wheel load, *σ* is the stress and *E* is elastic constant called the Young's modulus. In our research, five embedded concrete strain gauges are employed to obtain traffic-induced dynamic pavement strain response time histories.

As summarized in Sun *et al.*, different categorization schemes have been adopted in recent vehicle classification studies [28]. According to a Federal Highway Administration (FHWA) standard, vehicles are categorized into the six classes: passenger cars, motorcycles, buses, other 2-axle 4-tire vehicles, single-unit 2-axle 6 tire or more trucks, and combination trucks. In our research, a similar categorization scheme was defined to sort vehicles into the five classes (Table 1): small vehicles, medium trucks, buses/large trucks, 3-axle trucks, and combination trucks. The small vehicle class includes motorcycles if the corresponding record strain signal exceeds the ambient noise level.

## 3. Preliminary Experiments

### 3.1 Description of the experiments

The experimental plan for field testing covered a wide range of pavement responses as a function of magnitude of load (empty, intermediate and fully loaded) and speeds (5, 20, 30, 40 and 60 km/h). A two axle truck of known weight was used in the study. The accuracy of the corresponding vehicle classification parameters will directly affected the accuracy of the classification. For the purposes of examining capture the accuracy of vehicle parameters, a two axle truck of known axle distance and vehicle length was used in the research. In order to examine measurement repeatability, every experiment with the same velocity and the location across the pavement was replicated 10 times.

### 3.2 The calculation of vehicle parameters

**Figure 7.**
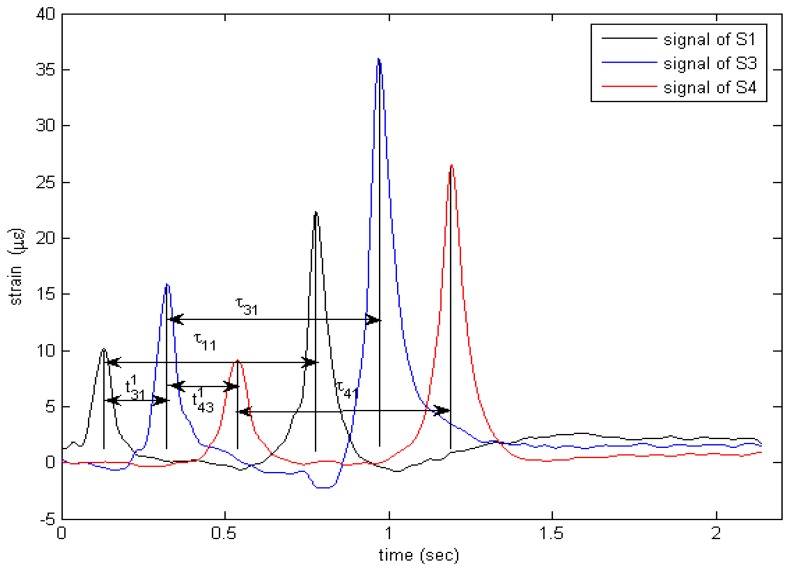
Vehicle classification using multiple embedded strain gauge sensors.

To get other vehicle parameters, an accurate speed should be measured first. Figure 7 shows the real response curves of sensors S1, S3, S4 when a 2-axle truck crosses them. A Matlab program is applied to each strain time history to automatically identify the basic parameters: *N* (Number of peak each curve) corresponding to number of axle, *v* (speed of vehicle passing over sensor), *WB* (distance between adjacent axle). Since the distance between sensors S1 and S3 or S3 and S4 is known, and this distance is far greater than the tire's contact length, for a 2-axle vehicle, the speed can be calculated by the formula below:

(2) $$\begin{array}{l} v=\left(v_{1}+v_{2}\right)/2\\ \text{where}\;v_{1}=\frac{s/2}{t_{31}^{1}};v_{2}=\frac{s/2}{t_{43}^{1}}. \end{array}$$

where s/2 is the distance between S1 and S3 or S3 and S4 (see Figure 6); *v*_1_ is the velocity measured by S1 and S3; *v*_2_ is the velocity measured by S3 and S4; $t_{31}^{1}$ and $t_{43}^{1}$ is the time interval between the first peak of sensor S1 and S3, S3 and S4, respectively (see Figure 7.). To obtain a more accurate speed, we take twice the average speed of measurements [refer to Equation (2)].

With the measured speed, the wheelbase (axle distance) of the vehicle can be measured using the following equation:

(3) $$\begin{array}{l} WB=(WB_{1}+WB_{2}+WB_{3})/3\\ \text{where}\;WB_{1}=v\cdot \tau_{11};WB_{2}=v\cdot \tau_{31};WB_{3}=v\cdot \tau_{41}. \end{array}$$

In the case of a vehicle with more than two-axles there would be a larger number of wheelbase distances. Specifically there are *N-1* wheelbase distances. Using the wheelbases and the number of axles, the vehicle can be classified using any standard classification scheme such as Table 1.

### 3.3 Variables that affect a vehicle classification

There are many factors affecting the operation of a classification. The Department of Transportation classifies a vehicle depending on the number of axles and the space between them. In order to measure the wheelbase some knowledge of the speed is necessary as indicated by the Equation (2). The accuracy measure time is very important in the estimation of speed and wheelbase. A potential source of error in the estimation of speed is the uncertainty in measurements. A small error in measurement of distance *s*/*2* between sensor S1 and S3 or S3 and S4 will introduce a larger error in the estimation of a vehicle's speed.

Errors in wheelbase estimation can be caused by the vehicle when its speed is not constant while the vehicle drives over the sensor. Acceleration of the vehicle will cause the wheelbase to seem smaller, while deceleration will cause the wheelbase to seem larger according to the classification system. Usually a classification system is placed at certain points in the highway with traffic signs that state that the speed should remain constant.

### 3.4. Two-axle truck experimental results and discussion

**Figure 8.**
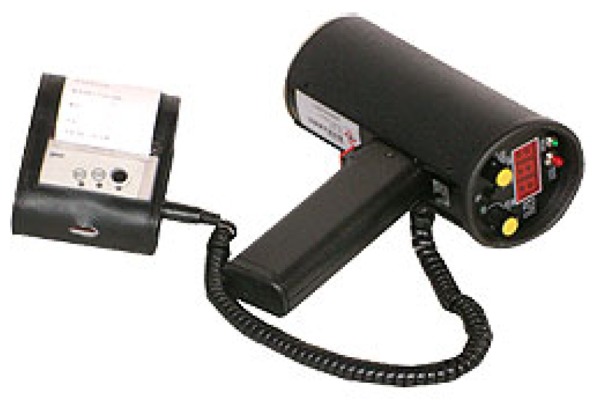
CSR-68 Speed radar gun.

Classification depends mostly on the accuracy in estimating the speed. Since the speedometer of a car is not sufficient accurate, it cannot be used as a speed reference. In order to acquire the system of measurement accuracy, the measurement results were compared with the results from a radar gun (Figure 8). CSR-68 Speed Radar Gun measuring accuracy is 0.1 km/h. A two-axle truck was used in this study.

Table 2 gives the experimental wheelbase measurement results. The wheelbase of the two-axle test truck was 6.67 m. The following data was obtained this vehicle with different speeds repeated 10 times. Again s/2 is the distance between the sensors S1 and S3 or S3 and S4 (cf. Figure 6). When the speed is known, the wheelbase may be calculated with Equation (3). The precision of the calculations is four decimal places; however in the Table below, to save space only the first two decimal places are shown.

The data in Table 2 show that the system is able to accurately estimate the speed and the wheelbase using multiple strain gauge sensors. Thus the use of multiple strain gauge sensors placed at different locations (refer to Figure 6) can decrease the uncertainties in measuring speed and wheelbase.

## 4. Description of SVM fusion classification

A classification based on a fixed wheelbase and number of wheelbases often leads to low accuracy. Since the patterns seemed to have a lot of overlap between them, this necessitated the use of SVMs pattern recognition and classification techniques to distinguish between vehicle groups.

### 4.2. Support vector classification

The SVM is a pattern recognition technique that is reported to be an excellent universal statistical learning machine with superior classification abilities [18]. The SVM classifies by mapping the data from an input space with an appropriate kernel function into a high dimensional feature space (hypothesis space) where a linear decision rule can be found based on observing the principle of maximizing the margin [19]. The SVM generates a function from labeled training data and predicts the output type of an unclassified novel input. The solution provided by SVM is said to always be optimal because of the absence of local extremes [20]. The method is structured in such a way that only a part of the training data, called support vectors, is used during training, hence avoiding computational complexity and providing better generalization. A detailed description of the general concept of SVMs is given by Burges [29], and Schölkopf and Smola [30]. This literature was the base for the choice of support vector machines for classification by researchers. A brief summary of SVM theory is given below.

#### 4.2.1. Principle of Support vector Classification

Since an *N*-class decision problem can be decomposed into a set of two-class problems we will firstly concentrate here on a two-class problem.

If a given training set is denoted as(*x_i_*, *y_i_*)*_i =_*_1,…,_*_n_*, where *x_i_* is an input vector, *y_i_* is its label and *n* is the number of training data, then a SVMs classification model in a high-dimension feature space can be represented as follows:

(4) f(x)=sgn(<w,ϕ(x)>+b)

where, *w* is a weight vector, *b* is a bias, *ϕ*(*x*) is a nonlinear mapping from the input variable into a high dimension feature space <, > denotes the dot product.

The optimal classification hyperplane can be obtained by the following primal formulation:

(5) $$\begin{array}{lll} \min_{w,b,\zeta } & \frac{1}{2}||w|{|}^{2}+C\sum_{i=1}^{n}\zeta_{i} & \\ s.t & y_{i}(<w,\phi (x)>+b)\geq 1-\zeta_{i} & i=1,\ldots ,n\\ & \zeta_{i}\geq 0 & i=1,\ldots ,n \end{array}$$

where $\frac{1}{2}||w|{|}^{2}$ controls the complexity of the model, *ζ_i_* is a slack variable measuring the error on *x_i_*, *C* is a regularization parameter, when determines the trade off between the empirical error and the complexity of the model. A Lagrangian function corresponding to (5) can be described as follows by introducing Lagrange multiplies *a_i_* ≥ 0 and *γ_i_* ≥ 0.

(6) $$L=\frac{1}{2}||w|{|}^{2}+C\sum_{i=1}^{n}\zeta_{i}-\sum_{i=1}^{n}\alpha_{i}[y_{i}(<w,\phi (x_{i})>+b)-1+\zeta_{i}]-\sum_{i=1}^{n}\gamma_{i}\zeta_{i}$$

Based on Karush-Kuhn-Tucker (KKT) optimality conditions:

$$\begin{array}{c} \nabla_{w}L=0\\ \nabla_{b}L=0\\ \nabla_{\zeta_{i}}L=0 \end{array}$$

We can get the following relations:

$$\begin{array}{l} w=\sum_{i=1}^{n}\alpha_{i}y_{i}\phi (x_{i})\\ \sum_{i=1}^{n}\alpha_{i}y_{i}=0 \end{array}$$

And the box constraints:

$$\begin{array}{cc} 0\leq \alpha_{i}\leq C, & \forall i=1,\ldots ,n. \end{array}$$

Plugging the latter equations in the Lagrangian, we derive the formulation of the dual problem. Hence the SVM classification becomes a Quadratic Programming (QP) problem:

(7) $$\begin{array}{cc} \underset{a_{i},i=1,\ldots ,n}{\min} & \frac{1}{2}\sum_{i=1}^{n}\sum_{j=1}^{n}\alpha_{i}\alpha_{j}y_{i}y_{j}k(x_{i},y_{j}) \end{array}-\sum_{j=1}^{n}\alpha_{i}$$

(8) $$\begin{array}{lll} \text{s}.\text{t}. & \sum_{i=1}^{n}\alpha_{i}y_{i} & \\ & 0\leq \alpha_{i}\leq C, & \forall i=1,\ldots n. \end{array}$$

From the solution of the dual, only the data that corresponding to non-zero values of *α_i_* work, and they are called the support vector (SV). Equation 4 can then be represented as in Equation 9 and the decision function that separates training vectors into classes in the input space is:

(9) $$d(x)=\mathrm{sgn}(f(x))=\mathrm{sgn}(\sum_{i=1}^{n}\alpha_{i}y_{i}k(x_{i},x)+b)$$

where *k*(*x_i_*,*x*)=< *ϕ*(*x_i_*), *ϕ*(*x*)> is the kernel function [21].

#### 4.2.2. Support vector classification training

The algorithm used by the SVM scans the training data and chooses from the hypothesis space a function that can fit the data with minimum error. The learning problem is solved through trading off between training error and the complexity of the hypothesis space. Training is conducted through finding the optimal classification function by solving the following regularization problem:

(10) $$\underset{f\in H}{\min}\frac{1}{n}\sum_{i=1}^{n}V(f(x_{i}),y_{i})+\lambda {\left\|f\right\|}_{k}^{2}$$

The term *λ* is the regularization parameter and ${\left\|f\right\|}_{k}^{2}$ is the norm of the hypothesis space [19]. The function *V* is called the loss function which measures how good the prediction function *f*(*x*) is in comparison to the training truth *y_i_*. The loss function used for classification purposes is a non-negative function of the form *V*(*f*(*x_i_*),*y_i_*) = max{(1–*y_i_f*(*x_i_*)),0}. The penalty to errors is used to control regularization, hence using the definition of $C=\frac{1}{2n\lambda }$, the training problem becomes:

(11) $$\underset{f\in H}{\min}\frac{1}{n}C\sum_{i=1}^{n}V(f(x_{i}),y_{i})+\frac{1}{2}{\left\|f\right\|}_{k}^{2}$$

#### 4.2.3 Multi-class Support Vector Machines

SVMs were originally designed for binary classification. From Table 1 we can see vehicle classification is a multi-class problem with more than two types. For the binary pattern recognition problem, the support vector was introduced in Section 4.2. In this section, we will review two popular SVM multi-class methods: One-Against-All (OAA) [22] and One-Against-One (OAO) [23].

The earliest used implementation for SVMs multi-class classification is probably the OAA method [24]. It constructs *k* SVMs models where *k* is the number of classes. The *i*th SVMs is trained with all of the examples in the *i*th class with positive labels, and all other examples with negative labels. Thus given *l* training data (*x_i_*, *y_i_*)*_i=_*_1,…_*_l_*, where *x* ∈ *R_n_* and *y_i_* ∈ {1,…,*k* is the class of labels (i.e., vehicle classes), the *i*th SVMs solves the following problem:

(12) $$\begin{array}{ll} \underset{w^{i},b^{i},\zeta^{i}}{\min} & \frac{1}{2}{\|w^{i}\|}^{2}+C\sum_{j=1}^{l}\zeta_{j}^{i}\\ & {\left(w^{i}\right)}^{T}\phi (x_{j})+b^{i}\geq 1-\zeta_{j}^{i},\text{if}\;y_{j}=i,\\ & {\left(w^{i}\right)}^{T}\phi (x_{j})+b^{i}\leq 1-\zeta_{j}^{i},\text{if}\;y_{j}\neq i,\\ & \zeta_{j}^{i}\geq 0,j=1,\ldots l. \end{array}$$

where the training data *x_i_* are mapped to a higher dimensional space by the function *ϕ* and *C* is the penalty parameter.

Minimizing $\frac{1}{2}{\left\|w^{i}\right\|}^{2}$ means that we would like to maximize 2/ ǁ*w^i^*ǁ^2^, the margin between two groups of data. When data are not linear separable, there is a penalty term $C\sum_{j=1}^{l}\zeta_{j}^{i}$ which can reduce the number of training errors. The basic concept behind SVMs is to search for a balance between the regularization term $\frac{1}{2}{\left\|w^{i}\right\|}^{2}$ and the training errors.

After solving (12), there are *k* decision functions:

$$\begin{array}{c} {\left(w^{1}\right)}^{T}\phi (x)+b^{1},\\ \vdots \\ {\left(w^{k}\right)}^{T}\phi (x)+b^{k}. \end{array}$$

We say *x* is in the class which has the largest value of the decision function:

(13) $$\text{class of d}(x)\equiv \arg\max x_{i=1,\ldots ,k}({\left(w^{i}\right)}^{T}\phi (x)+b^{i}).$$

In practice we solve the dual problem of (12), whose number of variables is the same as the number of data in (12). Hence *k l*-variable quadratic programming problems are solved.

Another major method is called OAO method. This method constructs $\frac{k\left(k-1\right)}{2}$ classifiers where each one is trained on data from two classes. For training data from the *i*th and the *j*th classes, we solve the following binary classification problem:

(14) $$\begin{array}{ll} \underset{w^{ij},b^{ij},\zeta^{ij}}{\min} & \frac{1}{2}/{\left\|w^{ij}\right\|}^{2}+C\sum_{m=1}^{l}\zeta_{m}^{ij}\\ & {\left(w^{ij}\right)}^{T}\phi (x_{m})+b^{ij}\geq 1-\zeta_{m}^{ij},\text{if}\;y_{m}=i,\\ & {\left(w^{ij}\right)}^{T}\phi (x_{m})+b^{ij}\leq 1-\zeta_{m}^{ij},\text{if}\;y_{m}=j,\\ & \zeta_{m}^{ij}\geq 0. \end{array}$$

There are different methods for doing the future testing after all $\frac{k\left(k-1\right)}{2}$ classifiers are constructed. After some tests, we decided to use the following voting strategy suggested in [25]: if the sign (*w^ij^*)*^T^ϕ*(*x_m_*) + *b^ij^* says *x* is in the *i*th class, then the vote for the *i*th class is added by one. Otherwise, the *j*th is increased by one. Then we predict *x* is in the class with the largest vote. The voting approach described above is also called the “Max Wins” strategy. In case those two classes have identical votes, though it may not be a good strategy, now we simply select the one with the smaller index.

In practice we solve the dual of (14) whose number of variables is the same as the number of data in two classes. Hence if on average each class has *l* / *k* data points, we have to solve *k*(*k*−1) / 2 quadratic programming problems where each of them has about 2*l* / *k* variables. The decision function for multi-class classification is:

(15) $$d(x)=\arg\underset{j}{\max}\left\{\sum_{i=1}^{m}\left|{\left(w^{ij}\right)}^{T}\phi \left(x_{m}\right)+b^{ij}\right|\right\}$$

That is, the class attribute of *x* is determined by sum of maximal distances to the optimal classification hyperplane.

There is a lot of software that uses SVMs to solve classification problems. In this research, the SVM-KM toolbox [26], which is a library of MATLAB routines, was used to handle multi-class problems. The use of SVM requires the preparation of two kinds of datasets, the training data with which to train a classification system and the test set on which to run the trained model. The collected data came from our developed test bench *in situ* (refer to Section 4.1).

The training of the SVM model basically involved the process of extracting the support vectors from the training data set. The support vectors defined the structure of boundary hyperplanes and the class hyperplanes after which the training data became useless. The so determined hyperplanes were stored for use of classification of novel data that had close similarity to the training data.

#### 4.2.4 Kernel selection

Prior to the training of the support vector machine model, the selection of an appropriate kernel function with which to map the data from the input space to the high dimension feature space, was of utmost importance. Lin *et al.* [27] proposed the radial basis function (RBF) to be the first choice for support vector machines practitioner, the reasons behind this claim were, first, the linear kernel is a special case of RBF; secondly, the sigmoid kernel behaves like RBF for certain parameters; thirdly the polynomial kernel has many parameters which makes their selection complex and fourthly and last, the RBF has less numerical difficulties. For these reasons, the RBF whose equation is shown below was used in this research:

$$\begin{array}{cc} K(x_{i},x_{j})=\exp(-\frac{{\left\|x_{i}-x_{j}\right\|}^{2}}{2\sigma^{2}}), & \sigma >0 \end{array}$$

#### 4.2.5 Cross-validation

The two parameters for the RBF model, the penalty *C* and the kernel function parameter *σ* have the best values for only one single problem. The values of these parameters should be known beforehand for a given problem prior to model training. The method used in the SVM-KM toolbox to find the values of these parameters is cross-validation. This cross-validation involves splitting the training data into subsets, leaving out one subset and training the classifier with different values of *C* and *σ* on the remaining subsets and measure the performance on the subset initially left out. The values of *C* and *σ* giving the highest cross-validation rates are chosen for training the classifier on the whole data set. If the best values of the parameters for a problem are not found, the obtained classifier will suffer from over fitting.

#### 4.2.6 Model training process

The *C* and *α* values obtained in the cross–validation were used for the training of the support vector machines. The training task involved the determination of the number of support vectors from each class, these vectors were used for the determination of weight vector, and the class and margin hyperplanes. When the training was over, a model file was created; this model file contained all the determined support vectors derived from the training data which were used in the classification of test data. After the determination of support vectors was done, the training data was not used anymore.

#### 4.2.7 Data Fusion Classification Strategies

During the past decades, the data fusion problem has been well researched. However, it is still an ongoing research area because of the promotion from advances in other fields. The synergistic use of overlapping and complementary data sources provides information that is otherwise not available from individual sources. Furthermore, multiple data sources can provide more robust performance due to the inherent redundancy. Therefore data fusion techniques of combining data from several data sources can yield higher accuracy and robustness than that achieved by single data source. The main architecture for fusion is centralized and distributed. In this paper, centralized and distributed architectures based on multiclass SVMs are introduced. The fusion schemes make full use of multiclass SVMs characteristic. The centralized fusion scheme is illustrated as the fusion Scheme A. the distributed fusion schemes is illustrated as the fusion Scheme B.

**Figure 9.**
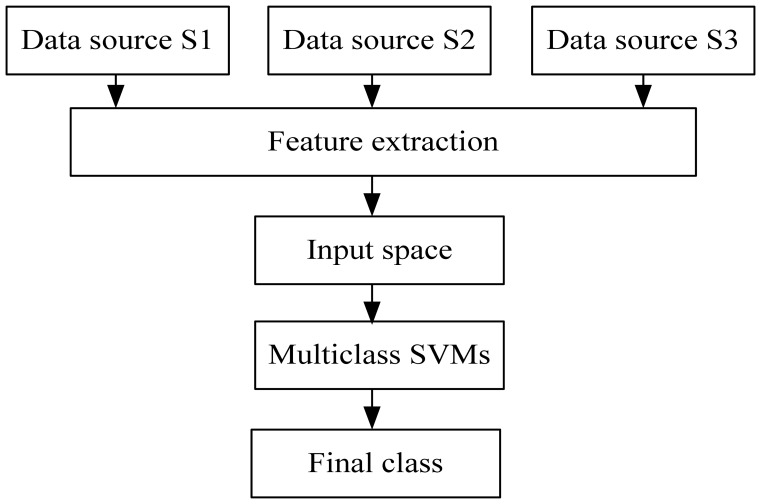
Data fusion architecture Scheme A.

In fusion Scheme A (see Figure 9), the features of all the sensor data sources are extracted and combined to form a single input space. Then the multiclass SVMs is trained and tested to create a decision maker.

**Figure 10.**
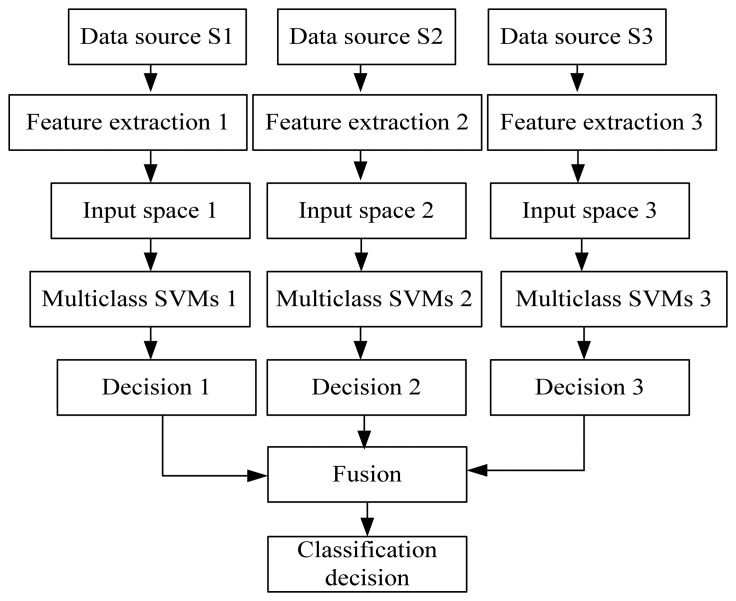
Data fusion architecture Scheme B.

In the fusion Scheme B (Figure 10), the features of every sensor data source are extracted and used to form an input space, respectively. Then the sub-Multiclass SVMs final classification decision is created. Here, the class attribute set is *k=* {1, 2, 3, 4, 5}, using the majority vote strategy, the final decision is:

(16) $$\begin{array}{l} d\left(x\right)=\arg\max\left\{V_{1},V_{2},\ldots ,V_{k}\right\},\\ V_{j}=\sum_{i=1}^{N}\delta_{ij},\delta_{ij}=\{\begin{array}{c} 1,d_{i}\left(x\right)=j\\ 0,d_{i}\left(x\right)\neq j \end{array}\\ \left(i=1,\ldots ,N;j=1,\ldots ,k\right) \end{array}$$

where *d* (*x*)is the final classification decision function, *V_j_* is comprised of the obtained votes of class *j* and *d_i_* (*x*) is the output of the *i*th Multiclass SVMs trained using the data from the *i*th sensor data source.

## 5. Experimental Results

Since freeways carry large volumes of vehicles, our data collection was installed on freeways to increase a possibility of having many vehicle types in the dataset, whilst using multiple strain gauge sensors for the measurements, thereby improving the accuracy of the classification parameters [17].

Data from five strain gauge sensors were collected *in situ* when a vehicle crossed the instrumental pavement. The panel was adopted as a test bed for periodic continuous monitoring. In 2005, during the 10 day data-collection experiment, through the data acquisition system, live data of pavement strain response induced by traffic was collected and stored. To thoroughly utilize the useful information, two features (number of axle and wheelbase) are extracted from the pavement strain time series (see Section 3.2). 602 different vehicle records were obtained. The records included a wide variety of vehicle types, ranging from 2-axle passenger cars to 6-axle semi-trailer trucks. Each instance is composed of 6 condition attributes (two features from each time series) and a class attribute (five states). In distributed schemes, the two features from one sensor data, added the class attribute, form an individual dataset [see (17)]. Therefore, three datasets from the corresponding three data sources are constructed. Following the classification scheme of Table 1, 602 records (Table 3) was manually labeled by artificial observation. The labeled records of each vehicle class are divided into a training set (50% of the records), validation set (25% of the records), and test set (25% of the records) of randomly selected records. Herein, this dataset was employed to develop and verify a strain-based vehicle classification approach.

(17) $$\begin{array}{l} \text{For S}1\left\{N_{1}^{1},WB_{1}^{1},WB_{2}^{1},WB_{3}^{1},WB_{4}^{1},WB_{5}^{1},\text{label}\right\},\\ \text{For S}3\left\{N_{2}^{3},WB_{1}^{3},WB_{2}^{3},WB_{3}^{3},WB_{4}^{3},WB_{5}^{3},\text{label}\right\},\\ \text{For S}4\left\{N_{3}^{4},WB_{1}^{4},WB_{2}^{4},WB_{3}^{4},WB_{4}^{4},WB_{5}^{4},label\right\},\\ \text{For S}1,\text{S}3,\text{S}4\;\text{combination}\\ \left\{N_{1}^{1},WB_{1}^{1},WB_{2}^{1},WB_{3}^{1},WB_{4}^{1},WB_{5}^{1},N_{2}^{3},WB_{1}^{3},WB_{2}^{3},WB_{3}^{3},WB_{4}^{3},WB_{5}^{3},N_{3}^{4},WB_{1}^{4},WB_{2}^{4},WB_{3}^{4},WB_{4}^{4},WB_{5}^{4},label\right\}, \end{array}$$

where the labels are numbered 1,2,3,4 and 5 to represent five classes vehicle (refer to Table 1.). All of the datasets contain a separate test set. A description of the data we used is given in Table 3.

The experiments in this paper are conducted on single sensor data and 3-sensor (S1, S3, S4) combination data sets, using the two different classifier algorithms (OAO and OAA). Two multiclass SVM algorithms are applied twice: on the individual sensor S3 data and 3-combination combination data. In both algorithms we used Gaussian kernels. To determine the values of *C* and *σ* we used cross validation on the training set. In all the experiments we set the value of *ζ* to 0.01. A summary of the results is shown in Table 4. The average classification accuracies of two types of Multiclass SVMs are given in columns D1, D3 and D4. Scheme I is the centralized fusion strategy. Scheme II is distributed strategies of data fusion based on majority-vote strategy. Table 4 shows that all the average classification accuracies of combined classifiers in fusion strategies outperform those of the classifiers not using fusion strategies. If the centralized data fusion strategy is used, the accuracy of the two types of Multiclass SVMs methods differs little (Column S.I in Table 4). However, in the distrusted data fusion Scheme II, the One-Against-One method has higher accuracy than One-Against-All method (Column S.II in Table 4).

## 6. Conclusions

A novel vehicle classification technique has been developed based on multiple pavement strains caused by moving traffic loads. Pavement strains are often used for monitoring the health of the road structure; here we regard it as a method of classification of vehicles. The main advantages of vehicle classification based on pavement strain are as follows: firstly, due to the installation of strain gauge sensors underneath the pavement, the sensor will not be affected by the impact load and has better durability; secondly, sensors would not be affected by bad weather; thirdly, the system may monitor day and night, especially at night so overloaded vehicles cannot escape monitoring; fourthly, since sensors are low-cost, it can provide a cheaper vehicle classification. It is an invasive measurement method, often installed my modifying the road, which is its main drawback.

A prototype system for measurement of pavement strain induced by moving vehicle traffic was developed and built to collect data for verifying and testing the feasibility and performance of the system. The estimation of the speed is very important, as it often affects the accuracy of the estimated vehicle wheelbase. In this research multiple strain sensors were used to measure speed from the average, so to a certain extent, estimation speed errors caused by acceleration and deceleration of vehicles will be eliminated. As an added benefit, multiple sensors can also expand the scope of measurements, providing more vehicles feature parameters.

The overlap of vehicle classification feature parameters belonging to different classes suggested the need to use a pattern recognition technique for separating vehicles into different groups. As an application of machine learning the support vector machines (SVMs) multi-class model was used for this purpose. To improve classification accuracy and robustness centralized and distributed fusion schemes based on two popular SVMs multi-class algorithms were used as fusion multiple sensor data. Comparison of experimental results shows the OAA and OAO methods with distributed fusion strategies are more suitable for practical use. In the paper, only two features are extracted from the pavement strain data and used to train SVM classifier. To improve classification accuracies and robustness, more features (such as vehicle length, axle load etc.) from strain time series to train SVM classifier will be studied.

## Figures and Tables

**Table 1.** Definition of Vehicle Classes.

**Vehicle type**	**Notation**	**Number of axles**	**Distribution of axles^1^**	**Vehicle types**	**FHWA vehicle categories**
Small vehicles	C1	2	1F+1R	Passenger car, Minivan, van, SUV, pickup truck	Passenger car, other 2-axle 4-tire vehicles
Medium trucks	C2	2	1F+1R	Medium single-unit 2-axle truck	Other 2-axle 4-tire vehicles
Buses/Large trucks	C3	2	1F+1R	Buses, large single-unit 2-axle trucks	Bus, single-unit 2-axle, 6-tire or more truck
3-axle trucks	C4	3	1F+2R	Single-unit 3-axle trucks	single-unit 2-axle 6-tire or more trucks
Combination trucks	C5	3-6	1F+1M+1R 1F+2M+1R 1F+1M+2R 1F+2M+2R 1F+2M+3R	Semi-trailer, truck, trucks with trailer	combination trucks

Note: 1.F-Front, M-Middle, R-Rear. (For instance: 1F+1R indicate a vehicle with one front axle and one rear axle.)

**Table 2.** The wheel base measurement using the proposed system.

**Number**	*τ*_11_**(sec)**	*τ*_31_**(sec)**	*τ*_41_**(sec)**	*v*_1_**(m/s)**	*v*_2_**(m/s)**	*v***(m/s)**	**WB(m)**	**Actual WB (m)**	**WB Error**
1	0.54	0.52	0.53	12.24	12.44	12.34	6.58	6.67	-1.3%
2	0.42	0.42	0.42	15.27	15.19	15.23	6.45	6.67	-3.2%
3	0.45	0.45	0.45	14.01	14.21	14.11	6.39	6.67	-4.1%
4	0.51	0.52	0.52	13.34	13.14	13.24	6.82	6.67	0.7%
5	0.43	0.43	0.43	15.11	14.91	15.01	6.49	6.67	-2.6%
6	0.59	0.59	0.59	11.28	11.34	11.31	6.72	6.67	-0.7%
7	0.51	0.51	0.51	12.79	12.96	12.85	6.59	6.67	-1.1%
8	0.54	0.54	0.54	12.87	12.67	12.77	6.88	6.67	1.7%
9	0.61	0.61	0.61	10.75	10.95	10.85	6.61	6.67	-0.8%
10	0.52	0.52	0.52	12.75	12.95	12.85	6.69	6.67	0.3%

**Table 3.** Numbers of Labeled Records.

**Vehicle type**	**Total**	**Training set**	**Validation set**	**Test set**
*C_1_*	72	36	18	18
*C_2_*	68	34	17	17
*C_3_*	174	88	42	42
*C_4_*	84	42	21	21
*C_5_*	204	102	51	51

**Table 4.** Comparison of Classification Results.

**Multiclass SVMs**	**Single sensor data source**			**S.I**	**S.II**

	**D1**	**D2**	**D3**		
One-Against-All (%)	89.5	92.3	90.9	94.6	95.5
One-Against-One (%)	91.4	91.8	91.2	94.4	96.4

Note: D1, D2, D3: Sensor S1 data, Sensor S3 data, Sensor S4 data; S.I, S.II: Scheme I, Scheme II.
